# Atypical Mycobacterial Infection Mimicking Psoriasis in an Elderly Patient: Diagnostic Challenges and Management

**DOI:** 10.7759/cureus.52139

**Published:** 2024-01-11

**Authors:** Muhammad Arshad, Aakash Kumar, Muaz Shafique Ur Rehman, Piere R Tito Rodriguez, Giustino Varrassi

**Affiliations:** 1 Rheumatology, National Hospital and Medical Centre, Lahore, PAK; 2 Medicine, Shaheed Mohtarma Benazir Bhutto Medical College, Karachi, PAK; 3 Internal Medicine, Jinnah Hospital Lahore, Lahore, PAK; 4 Medicine, University of the Valley, La Paz, BOL; 5 Pain Medicine, Paolo Procacci Foundation, Rome, ITA

**Keywords:** infection, mycobacterial, rheumatoid arthriitis, arthritis, psoriasis

## Abstract

Atypical mycobacterial infections can have diverse clinical presentations, frequently resulting in diagnostic challenges. This study examines an uncommon atypical mycobacterial disease in an older patient, initially misidentified as psoriasis, emphasizing the difficulties in diagnosing and managing the condition. A 70-year-old male patient arrived at the dermatology department at National Hospital and Medical Centre, Lahore, with a persistent, dry, red rash mainly affecting his arms and legs. The patient had a medical background of diabetes mellitus and hypertension. Initial clinical diagnosis suggested psoriasis based on the appearance and patient's age. The patient was started on conventional psoriatic therapies. However, there was only a slight improvement, which led to the need for additional inquiry. Skin biopsies were conducted, uncovering the presence of granulomatous inflammation. Following cultures and polymerase chain reaction (PCR) tests, the presence of atypical mycobacteria was established. Subsequent laboratory tests eliminated the possibility of tuberculosis (TB) and other prevalent diseases. The conclusive diagnosis was an unusual mycobacterial infection, which posed a challenge due to its clinical similarity to psoriasis. The patient was treated with antibiotics appropriate to the mycobacterial species discovered. The duration of the treatment spanned six months, resulting in a notable amelioration of the skin lesions. The patient is being closely observed for any potential reoccurrence. This case highlights the significance of including atypical mycobacterial infection as a possible diagnosis for persistent dermatological problems, particularly in persons with impaired immune systems. It emphasizes the need for biopsy and culture in unusual situations of suspected psoriasis. This instance also demonstrates the intricacies associated with managing older patients with multiple concurrent medical issues. Unusual mycobacterial infections can imitate conventional skin disorders such as psoriasis, presenting considerable difficulties in diagnosis. Having a strong suspicion and doing relevant laboratory tests are essential for achieving an accurate diagnosis and efficient treatment.

## Introduction

In dermatology, clinical situations are often challenging to diagnose, especially when common skin disorders are mistaken for rare infections. An example is when mycobacterial diseases have an unusual appearance that resembles more typical skin conditions like psoriasis [1]. This case report examines a fascinating instance of an uncommon mycobacterial infection in an older patient, initially misdiagnosed as psoriasis. It highlights the difficulties in making an accurate diagnosis and managing the complexity of the condition. Non-tuberculous mycobacteria (NTM) are responsible for atypical mycobacterial infections, which are being more acknowledged in clinical settings. Contrary to tuberculosis, these illnesses are not commonly linked to individual transmission [1]. Instead, they are obtained by exposure to the environment. The prevalence of NTM infections has been increasing, primarily due to advancements in diagnostic procedures and heightened awareness among doctors. Nevertheless, determining the actual frequency is challenging because of inadequate reporting and the varied clinical presentations of these diseases [2].

Psoriasis is a persistent, inflammatory skin disorder with red, scaly patches. It impacts around 2-3% of the population and is characterized by its fluctuating nature. Psoriasis is frequently considered a primary possible diagnosis in individuals with chronic skin complaints because it is common and has distinct features [1]. This presents a diagnostic dilemma when managing unusual illnesses that may exhibit comparable clinical characteristics. Distinguishing between psoriasis and atypical mycobacterial infection based solely on clinical criteria might be challenging. Both illnesses may manifest as red patches of skin and flaking. Nevertheless, it is crucial to meticulously consider inconspicuous clinical distinctions, the patient's medical background, and other circumstances that increase the risk [2]. Diagnosis primarily relies on laboratory studies, such as skin biopsies, histological examination, and microbiological cultures.

Although primarily known for their skin-related symptoms, atypical mycobacterial infections can also significantly affect the musculoskeletal system, leading to systemic ramifications. A vital consequence that can arise is the onset of arthritis, especially in older people or those with weakened immune systems [3]. This form of arthritis commonly manifests as a persistent, slow-moving condition, which can result in significant delays in diagnosis. The development of the disease requires the spread of the mycobacterium through the bloodstream or the direct expansion from nearby infected tissues to the joints. The clinical manifestation often involves a single joint, with the knees and hips being the most frequently impacted joints [3]. From a radiological perspective, the affected joints may exhibit indications that align with inflammatory arthritis, such as a reduction in joint space, bone tissue erosion, and joint deformation in long-standing cases. Nevertheless, these radiographic observations are not exclusive to a particular disease. They can imitate other types of arthritis, contributing to the intricacy of diagnosis. Histopathological analysis of synovial tissue and microbiological cultures remains essential for establishing a precise diagnosis. Arthritis in the skeletal system emphasizes the widespread nature of atypical mycobacterial infections [3]. It highlights the importance of thoroughly assessing patients who have these infections. This is necessary to identify and treat these potentially disabling complications quickly. The diagnostic process is marked by problems such as collecting samples, extended culture durations, and the interpretation of findings [4].

A comprehensive approach, including multiple disciplines, is necessary for managing atypical mycobacterial infections. Treatment protocols sometimes entail extended durations of antibiotic administration, wherein the selection of medications is informed by species identification and susceptibility testing. The discussion is around the intricacy of managing treatment in elderly individuals, who frequently experience comorbidities and are prone to adverse effects from medications. The study highlights the significance of considering unusual mycobacterial infections in patients exhibiting psoriasis-like lesions, especially when regular treatments are ineffective. This paves the way for a comprehensive case report that contributes to the current body of knowledge on unusual mycobacterial diseases and emphasizes the importance of a meticulous and unbiased approach in clinical diagnostics.

## Case presentation

This case report examines a 70-year-old male patient who initially exhibited dermatological symptoms resembling psoriasis but was ultimately identified with an unconventional mycobacterial infection. The complexity of diagnosing and controlling atypical mycobacterial infections in older adults is underscored by the convoluted case, which involves comorbidities of diabetes mellitus and hypertension, as well as rheumatological implications. The individual, a 70-year-old male with a medical background of diabetes mellitus and hypertension, arrived at the dermatology clinic of the National Hospital & Medical Centre Lahore. The patient presented with a persistent, dry, red rash, mainly on his arms and legs. The patient had a noteworthy medical history of the indicated chronic illnesses, which were controlled with pharmacological treatment. His familial background, lifestyle variables, and any prior occurrences of such symptoms were also recorded. Upon inspection, the patient displayed clearly defined, dry, red patches on his limbs. The plaques exhibited little tenderness and showed no indications of pustules or systemic involvement. Significantly, there was an absence of joint swelling or deformity at this particular period. The initial evaluation prioritized the examination of the patient's dermatological characteristics, taking into account their age and symptoms.

**Figure 1:**
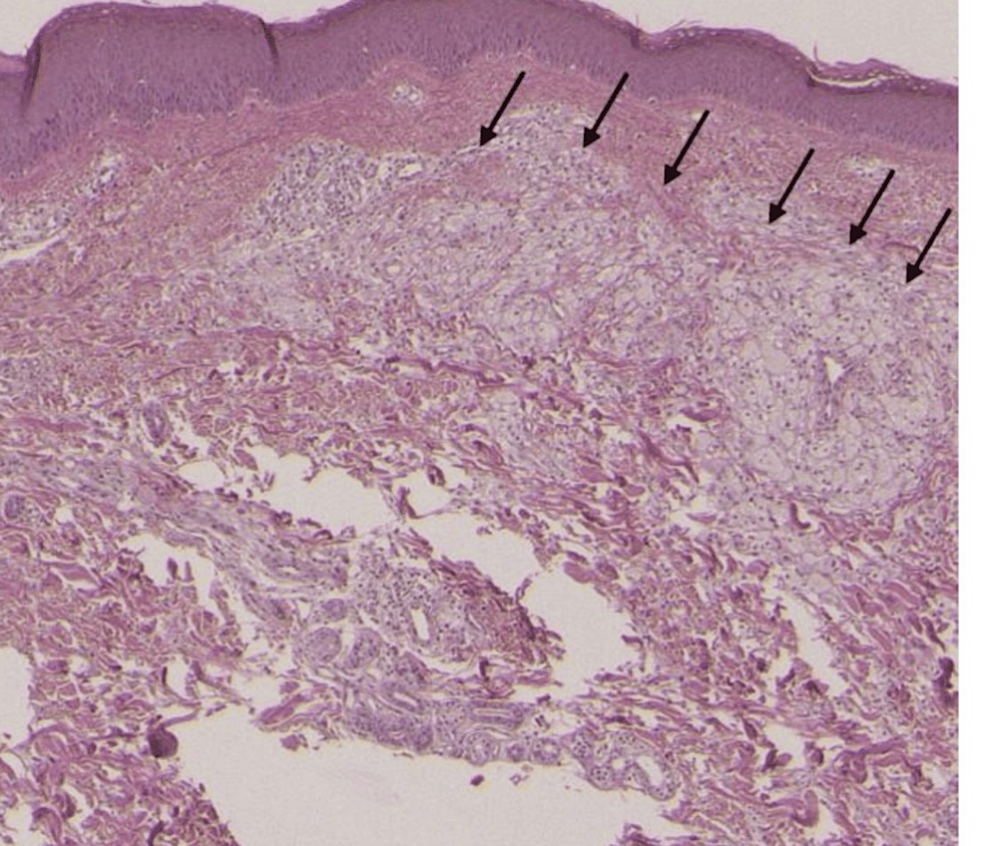
Punch Biopsy Showing Granulomatous Inflammation Stain: Hematoxylin and eosin stain (H&E), epidermis and dermis with 10 × magnification.

The initial symptoms and examination findings resulted in a provisional diagnosis of psoriasis. The treatment began with the application of topical corticosteroids and moisturizers. Nevertheless, the patient's limited reaction to these therapies sparked concerns, leading to a reassessment of the diagnosis. Further inquiries included skin biopsies taken from the impacted regions. The histopathological examination showed the presence of granulomatous inflammation, which is an atypical sign of psoriasis, as shown in Figure 1.

Therefore, additional diagnostic investigations are required. The patient's diagnostic path took a critical turn when polymerase chain reaction (PCR) testing detected atypical mycobacteria. Simultaneously, radiographic evaluations were performed to assess potential musculoskeletal involvement, considering the patient's age and the recognized tendency of atypical mycobacterial infections to impact the bones and joints. Considering the patient's age and the fact that mycobacterial infections are known to affect the entire body, it was determined that a thorough examination by a rheumatologist was necessary. This encompassed a comprehensive evaluation, which involved a collaborative inspection, radiographic imaging, and blood testing to evaluate the presence of inflammation and joint deterioration indicators. The objective was to eliminate or verify any rheumatological consequences, such as arthritis, that may occur due to the mycobacterial infection. Table 1 shows detailed treatment options and timeline.

**Table 1:** Detailed Treatment Options and Timeline

Stage	Treatment Options	Rationale
Initial Assessment	Topical Steroids and Emollients	Psoriasis is a prevalent skin ailment; the earliest symptoms indicated its probable presence. Steroids are utilized for their anti-inflammatory properties.
Lack of Improvement	Systemic Methotrexate and Topical Calcineurin Inhibitors	The escalation of symptoms necessitated a more assertive approach. Methotrexate is used to suppress the immune system in cases of suspected psoriasis.
Biopsy Results	Discontinuation of Methotrexate, Initiation of Antibiotics	The presence of granulomatous inflammation suggests that there may be an infectious cause termination of immunosuppressive treatment and commencement of antimicrobial protection.

At this point, the range of possible diagnoses was broadened to encompass skin problems and disorders related to the joints and connective tissues. Considering the systemic character of the diagnosed infection, the potential presence of psoriatic arthritis, rheumatoid arthritis, or other types of infectious arthritis was taken into account. The definitive diagnosis of an atypical mycobacterial infection was established by combining dermatological observations, histopathological analysis, and PCR test results and eliminating other possible diagnoses. This diagnosis highlighted the difficulty of detecting unusual manifestations in older patients, especially those with pre-existing medical conditions. The management strategy employed a customized approach, considering the condition's dermatological and possible rheumatological elements. The patient commenced a course of antibiotics tailored to the diagnosed mycobacterial species. The selection of antibiotics and the length of therapy (six months) were decided according to the sensitivity patterns of the species and the patient's general health condition. In addition, the patient's diabetes and hypertension were carefully controlled to enhance his immunological response and overall well-being. The patient's reaction to the treatment was carefully observed, with frequent check-ups to evaluate the effectiveness of the treatment plan and address any potential adverse effects. Noticeable enhancement was noticed in the skin lesions during the treatment period. In addition, the patient underwent ongoing assessment to detect any indications of rheumatological problems, such as arthritis, that may require further therapies. Table 2 shows detailed timeline and response assessment.

**Table 2:** Timeline and Response Assessment

Time Point	Treatment Plan	Observations
Initial Presentation	Topical Steroids and Emollients	Mild improvement in rash, but persistent dryness.
2 Weeks	Continued Topical Treatment	Limited response, progression of lesions noted.
4 Weeks	Methotrexate Initiation	Mild improvement in redness, persistence of dryness.
8 Weeks	Topical Calcineurin Inhibitors Added	Slight reduction in redness, no significant change in dryness.
Biopsy Results	Antibiotics Initiated	Discontinuation of Methotrexate. Antibiotics started based on biopsy results.
12 Weeks	Antibiotic Regimen	Gradual improvement observed, reduction in redness.
16 Weeks	Continuation of Antibiotics	Further reduction in redness and improvement in dryness.
24 Weeks	Completion of Antibiotic Course	Notable amelioration of skin lesions, rash nearly resolved.

## Discussion

Atypical mycobacterial infections encompass a range of clinical manifestations, emphasizing the need to consider the current case in the context of existing literature. The occurrence of an unusual mycobacterial condition that imitates psoriasis in an elderly patient highlights the wide range of variations found in these types of diseases. The existing body of literature presents various clinical symptoms, underscoring the importance of increased awareness among healthcare professionals. Our described case exhibits similarities with several published cases, notably regarding the initial misdiagnosis [1]. Identifying atypical mycobacterial infections is frequently challenging due to misdiagnoses caused by similar symptoms to other dermatological disorders. A study showed a Mycobacterium avium complex infection initially misdiagnosed as eczema [1]. This case highlights the challenging nature of diagnosis, similar to what we experienced. The concurrence of symptoms among many mycobacterial species emphasizes the significance of a precise and prompt diagnosis. Additionally, the research emphasizes the importance of age as a determining factor in the clinical manifestation of atypical mycobacterial infections [2]. A retrospective review found more significant atypical mycobacterial diseases among older individuals, consistent with our case involving an elderly patient. The heterogeneity in clinical manifestations may be attributed to age-related differences in immune response and comorbidities, highlighting the need for a sophisticated approach to diagnosis and treatment [3]. Although our case has similarities with other studies, noticeable differences highlight the diversity within atypical mycobacterial infections. A vital deviation is the initial misdiagnosis as psoriasis, a situation less commonly documented in the literature. The majority of cases emphasize the occurrence of incorrect diagnosis associated with prevalent dermatological disorders such as eczema or fungal infections [3]. This disparity highlights the intricate and fluctuating nature of the clinical environment surrounding atypical mycobacterial diseases.

The diagnostic process in our case was characterized by significant difficulties, mainly arising from the initial misidentification of psoriasis. Contemplating this process of diagnosing the illness offers a valuable understanding of the complex characteristics of unusual mycobacterial infections [4]. A significant obstacle faced was the similarity between the skin symptoms and psoriasis. The erythematous plaques, scaling, and pruritus found in our patient closely matched the clinical criteria for psoriasis. Such imitation has been observed in the literature, as evidenced by a case of Mycobacterium marinum infection initially misdiagnosed as psoriasis [4]. The similarity of symptoms presents a challenging diagnostic situation, requiring a thorough assessment to distinguish between the two conditions. The patient's age played a role in the difficulties encountered during the diagnosis process in our case. The geriatric population frequently exhibits a wide range of dermatological disorders, and the presence of an atypical mycobacterial infection in our patient further contributed to the complexity of the diagnosis [5]. The weakened immune response and changed skin physiology that occur with aging affect how conditions appear clinically, making the diagnostic process more complicated. This is consistent with recent studies, which emphasized the differences in how mycobacterial skin infections manifest in different age groups [6]. Our case's delayed identification highlights the need for increased clinical suspicion, especially when encountering dermatological disorders that do not respond to traditional therapy. The initial customized treatment plan for psoriasis did not produce the anticipated outcomes, leading to a reassessment of the diagnosis. This observation aligns with recent literature findings, highlighting the significance of reevaluation in situations where initial interventions demonstrate ineffectiveness [6]. Despite its time-consuming nature, the iterative diagnostic method is essential for understanding the intricacy of atypical mycobacterial infections.

The diagnostic method was significantly influenced by histopathology, which provided essential information about the granulomatous inflammation typical of atypical mycobacterial infections. The skin biopsy played a crucial role in understanding the underlying disease, directing further investigations, and verifying the necessity for a thorough diagnostic evaluation [7]. The presence of granulomas in the biopsy sample suggested the involvement of mycobacteria, requiring additional analysis using microbiological tests. Diagnostic procedures, including cultures and PCR, have become essential for verifying the existence of atypical mycobacteria. Culturing facilitated the process of isolating and identifying the particular species of mycobacteria, hence aiding in developing tailored therapeutic therapies [6]. The diagnosis was further confirmed, and other possible infections were excluded by using PCR, which has a high sensitivity and specificity. The collaboration between histology and laboratory tests demonstrates the interconnectedness of diagnostic methods, underscoring their combined significance in deciphering intricate cases.

The selection of antibiotic treatment was determined based on the identification of the mycobacterial species. To ensure effective therapy, it is crucial to use antibiotic regimens specifically designed for the wide range of atypical mycobacteria. The susceptibility testing in our situation allowed for identifying a suitable antibiotic, enhancing therapeutic results [7]. The duration of treatment is of utmost significance in the management of atypical mycobacterial infections. Many illnesses' persistent and slow-developing nature requires an extended period of antibiotic treatment. Recent studies highlight the importance of longer treatment duration in improving lesions and preventing their reappearance [8]. The primary objective of this comprehensive treatment is to eliminate both the actively reproducing organisms and the latent forms, thus reducing the likelihood of a recurrence. Obstacles in antibiotic therapy encompass the possibility of adverse effects and interactions with the patient's preexisting medical issues [8]. It is crucial to closely monitor for adverse reactions and make necessary changes to the treatment plan to achieve the best therapeutic results while minimizing problems.

The patient will receive rigorous, extensive, long-term surveillance to identify possible recurrence or problems. Regular subsequent visits, followed by clinical and laboratory evaluations, will aid in promptly detecting any reemergence of mycobacterial activity [8]. Due to the persistent nature of atypical mycobacterial infections, it is crucial to closely follow patients over an extended period to ensure continued treatment effectiveness. Prolonged surveillance is essential not only for detecting the reappearance of a condition but also for managing potential aftereffects and safeguarding the overall health and welfare of the patient [9]. Comprehensive and continuing care necessitates a multidisciplinary approach, which includes dermatologists, infectious disease specialists, and other pertinent healthcare professionals. The interaction between rheumatological variables and dermatological manifestations complicates the diagnosis process, particularly in situations that mimic autoimmune disorders such as psoriasis [9]. Including rheumatological insights dramatically enhances the ability to distinguish between various diagnoses, leading to a more sophisticated comprehension of intricate cases. Given the autoimmune character of psoriasis, the rheumatological approach was essential in improving the diagnostic trajectory.

Gaining a comprehensive understanding of the complex connection between rheumatological disorders and dermatological symptoms promotes a well-rounded approach to patient management. Collaboration between dermatologists and rheumatologists is crucial when overlapping clinical presentations occur. This collaborative framework improves the precision of diagnoses and guarantees that patients receive thorough care that encompasses dermatological and rheumatological issues [10]. This instance highlights the significance of employing a multidisciplinary strategy when dealing with intricate dermatological issues. Collaboration between dermatologists, rheumatologists, and general practitioners is necessary to traverse the complex intricacies of atypical mycobacterial infections that resemble other dermatological disorders [9]. The diagnostic framework outlined in this case enhances clinical practices in dermatology by emphasizing the importance of a thorough assessment that includes histology, laboratory tests, and interdisciplinary consultations. The intricacy of this situation requires a transition from individualized specialty-focused methods to integrated care models [4]. Dermatologists should actively solicit rheumatological viewpoints in instances with autoimmune origins, and general practitioners should be watchful for unusual presentations that may require consultation with a specialist [5]. The collaborative nature of this mindset is in line with the changing patterns in healthcare, which advocate for a patient-focused, comprehensive, and scientifically supported method for dealing with intricate dermatological problems.

The assessment and management of the patient's quality of life are crucial factors in the diagnostic process and treatment. The era of misdiagnosis, characterized by uncertainty and inadequate remedies, may have caused psychological suffering [8]. Recognizing the patient's encounter during this duration is crucial for cultivating a therapeutic connection and attending to any enduring psychological repercussions. After receiving a precise diagnosis, the patient probably felt relief and fear. Comprehending the psychological elements of the diagnostic procedure is essential for customizing patient-centered therapy [1]. The healthcare team is responsible for delivering both efficacious medical intervention and emotional assistance and imparting knowledge to enable patients to manage their illnesses effectively. Hence, the patient's encounter should shape continuous deliberations on treatment compliance, anticipated outcomes, and coping strategies, guaranteeing a comprehensive approach to healthcare [10].

## Conclusions

To summarize, this case report sheds light on the complex process of diagnosing an unusual mycobacterial infection that initially presented as psoriasis in an older man with diabetes mellitus and hypertension. Diagnosing psoriasis, which first involved an incorrect diagnosis, progressed through skin samples, cultures, and PCR testing, ultimately accurately identifying atypical mycobacteria. This discovery challenges the traditional diagnostic approach. Following the customized administration of antibiotics and the cessation of immunosuppressive treatments, there was a significant improvement in the skin lesions within six months. This example highlights the crucial significance of a thorough and flexible diagnostic strategy, emphasizing the requirement for complete laboratory investigations, mainly when the diagnosis is ambiguous. In addition, incorporating rheumatological factors enhances the comprehension of intricate dermatological problems in elderly adults. This case offers valuable insights into the issues of diagnosing atypical mycobacterial infections, highlighting the importance of precise and prompt diagnosis for successful treatment outcomes as the medical community adapts to changing diagnostic landscapes.
